# Intelligent energy management of a novel non-isolated bidirectional dual-input single-output converter for electric vehicle applications

**DOI:** 10.3389/frai.2026.1950048

**Published:** 2026-10-29

**Authors:** Faridha Banu M. R., Jayapragash R.

**Affiliations:** School of Electrical Engineering, Vellore Institute of Technology, Chennai, Tamil Nadu, India

**Keywords:** bidirectional power transfer, intelligent energy management, interleaved boost topology, MODNN, multiport converter, regenerative braking, unified charging and discharging

## Abstract

For use in electric vehicle (EV) applications, this paper suggests a non-isolated bidirectional Dual-Input Single-Output (DISO) DC–DC converter with an intelligent energy management method. The proposed converter integrates photovoltaic (PV) and battery energy sources in a single power conversion structure, which results in fewer components, higher efficiency and bidirectional power flow capability. The converter is designed based on a two-phase interleaved boost topology with a relay-assisted reconfiguration stage that facilitates flexible source selection, battery charging/discharging and regenerative braking operation. A Modified Deep Neural Network (MODNN) based intelligent energy management strategy is proposed to enhance energy utilization and adaptive power sharing. The proposed MODNN modifies a conventional single-task feedforward DNN by introducing a shared-trunk, multi-head architecture that jointly predicts the discrete operating mode and the continuous converter duty ratios through a composite classification-regression loss function, benchmarked against recent machine-learning baselines (k-NN, a bagged neural-network ensemble, and LSTM) under identical training/test conditions. In the proposed MODNN framework, the PV voltage, PV current, battery state-of-charge, battery voltage, and load demand information are used to decide the optimal operating mode and power flow condition under different operating scenarios. The converter provides flexible power distribution between available energy sources, productive use of regenerative braking energy, uninterrupted load supply and effective battery management. The performance of the proposed converter is verified by MATLAB/Simulink simulations, and the converter topology is experimentally validated with a laboratory prototype of 500 W. The experimental results show that the five operating modes operate successfully with a peak efficiency of 96.7%, which validates the effectiveness of the proposed converter for EV energy management applications.

## 1 Introduction

Electric Vehicles (EVs) have emerged as a desirable alternative to conventional fossil fuel-based vehicles. This is due to a combination of factors such as the growing population of the world, increasing energy needs, rising oil and natural gas prices and depletion of limited supplies of fossil fuels. To tackle these challenges, there is an increasing interest in developing electric cars fueled by clean and renewable energy sources, offering a promising solution for sustainable and environmentally friendly transportation (Ehsani et al., 2010; Zhang and Mi, 2011). Photovoltaic cell (PV) stacks have emerged as a clean energy solution for EVs. PV cells convert sunlight directly into electrical power by means of photovoltaic reactions and thus represent a technologically advanced and environmentally friendly method of energy generation.

Their electricity output depends on the amount of sunlight. Generally, the random availability of solar energy is controlled by using Battery Energy Storage Systems (BESSs) to increase system efficiency and battery life (Peyman et al., 2011; Thounthong et al., 2009; Thounthong et al., 2010; Wang et al., 2011; Zandi et al., 2011). The conventional energy management techniques based on rule-based control, fixed-threshold logic, and classical maximum power point tracking (MPPT) algorithms typically exhibit low adaptability under rapidly changing operating conditions. For real-time energy management and power sharing applications, Artificial Intelligence (AI)-based techniques such as Artificial Neural Networks (ANNs), Deep Neural Networks (DNNs), Recurrent Neural Networks (RNNs), and machine learning-assisted predictive controllers have emerged as promising solutions (Dankar et al., 2025; Gupta et al., 2022).

Recent studies (Duarte et al., 2007; Krishnaswami and Mohan, 2009; Michon et al., 2004) have shown the effectiveness of neural network-based control techniques for PV-battery integrated energy systems. A NN-based energy management system for PV-battery microgrids can adaptively allocate power and balance state-of-charge levels under varying renewable energy conditions (Faraji et al., 2021; Koca et al, 2024). Similarly, NN-assisted predictive control strategies have been reported for PV-battery-EV systems, allowing improved bidirectional power flow management, renewable energy utilization and battery lifetime enhancement. Additionally, DNN-based control frameworks have been investigated for EV charging stations and hybrid renewable energy systems to enhance load management and operational efficiency under dynamic loading conditions. These evolutions show the increasing potential of intelligent learning-based approaches for next-generation EV energy management systems. More recently, artificial-neural-network-based fault-tolerant controllers have been reported for multi-source DC-DC converters combining photovoltaic and wind inputs for EV applications (Mohanty et al., 2026), and approximate-dynamic-programming-assisted neural network controllers have been demonstrated for universal converter structures in microgrid settings (Suresh et al., 2024), further confirming the effectiveness of learning-based control for compact, multi-input power-sharing architectures.

Despite these developments, the majority of published research concentrates on energy forecasting, charging optimization, or grid-level energy management; intelligent decision-making with compact multiport converter topologies that support photovoltaic power utilization, battery charging/discharging, regenerative braking, and adaptive source selection within a unified converter structure has received less attention. Furthermore, a lot of current solutions call for more components, numerous converter stages, or computationally demanding optimization algorithms that might not be appropriate for real-world EV applications. In this direction, a twin-delayed deep deterministic policy gradient (TD3)-based deep reinforcement learning controller was recently proposed for a bidirectional dual-input single-output converter supplying a constant-power load in a low-power EV application, achieving markedly faster settling and lower voltage error than conventional PI and super-twisting controllers (Hosseini et al., 2026); however, this model-free approach was developed for a battery/motor-drive configuration and does not incorporate photovoltaic hybridization or adaptive source-selection decisions, which the present work addresses.

This paper suggests a Modified Deep Neural Network (MODNN)-based intelligent energy management approach combined with a non-isolated bidirectional DISO converter for EV applications to overcome these drawbacks. In order to facilitate adaptive operating mode selection and intelligent power-sharing decisions among the various energy sources, the proposed MODNN framework makes use of photovoltaic power, battery state-of-charge, battery voltage, and load demand data. The researched method seeks to increase the operational flexibility of hybrid PV-battery EV systems, allow regenerative braking energy recovery, and improve energy utilization when combined with the suggested interleaved converter topology.

As seen in Figure 1a, integrating these various energy sources frequently requires controlling various voltage levels, which calls for the use of several DC-DC converters. Nevertheless, using separate converters for every energy source raises costs, complexity, and losses. To mitigate these challenges, the concept of multi-input DC-DC converters has been developed in the field of hybrid power systems, as shown in Figure 1b. The two primary kinds of multi-input converters discussed in this topic are isolated and non-isolated DC-DC converters.

**Figure 1:**
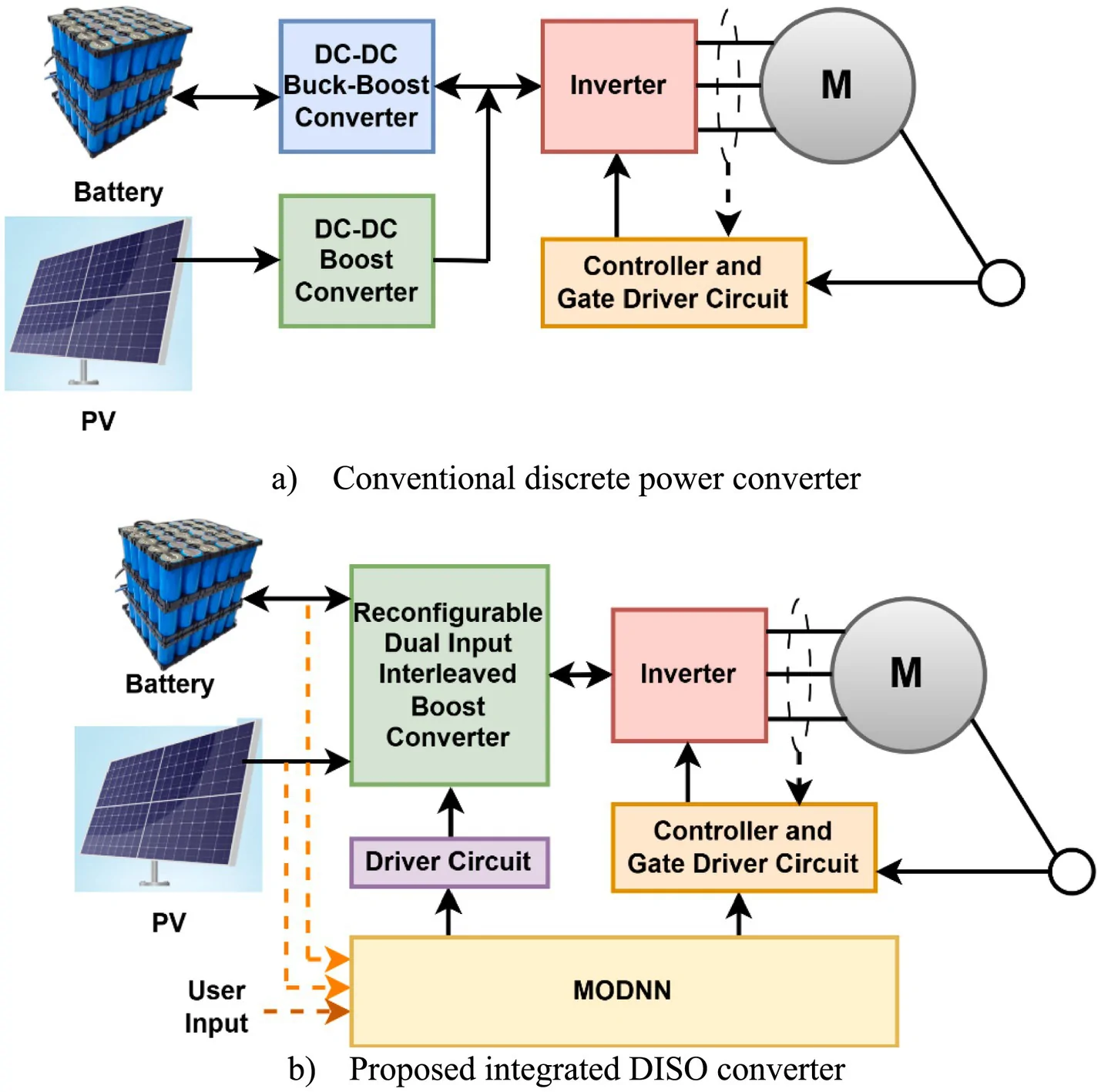
Block diagram.

In order to establish electrical isolation and impedance matching between the input and output sides, isolated converters (Tao et al., 2008; Tao et al., 2006; Tao et al., 2008; Ahmadi and Ferdowsi, 2012; Khasim et al., 2023; Narasipuram and Mopidevi, 2024) rely on high-frequency transformers and use leakage inductance to transmit power. Isolated converters are more complex and costly despite their efficacy since they need rectifiers, inverters, and transformer losses. The absence of transformer losses and the switching losses of more active switches boost the non-isolated DC-DC converter’s overall efficiency.

The system’s dependability is increased by using fewer parts and more straightforward control methods. Non-isolated multi-input converters, on the other hand, provide a workable solution for EV applications (Fesli and Ozdemir, 2024; Narasipuram and Mopidevi, 2025).

Although multiport and dual-input topologies have been extensively documented, the majority either require numerous converter stages or concentrate on unidirectional operation. Improved thermal, EMI, or high-gain characteristics have been highlighted in recent research (Narasipuram et al., 2025; Dezhbord et al., 2022; Faraji et al., 2021; Faraji et al., 2021), but they do not incorporate regenerative charging or straightforward source reconfiguration. On the other hand, our approach is innovative in that it enables EV-oriented hybridization of PV and battery sources by integrating interleaving, bidirectional battery charging, and relay-assisted source selection into a single converter with few components. Recent non-isolated multiport designs have likewise targeted EV-oriented hybridization, including bidirectional multiport converters for off-board EV charging stations that integrate photovoltaic and grid power (Mostafa et al., 2025), high-efficiency poly-input boost converters coupling solar and fuel-cell sources with a battery backup (Singh et al., 2024), and reconfigurable multiport converters unifying photovoltaic, EV and battery ports within a single structure (Khan et al., 2025). The converters under comparison (Dezhbord et al., 2022; Faraji et al., 2021; Faraji et al., 2021; Gaurav et al., 2023; Rostami et al., 2021; Saafan et al., 2023; Xu et al., 2021; Zhou et al., 2022; Jalilyan et al., 2024; Jalilyan et al., 2025) contain three or four ports and non-isolated constructions.

The suggested design achieves unified bidirectional energy management without the need for extra stages by integrating five functional modes with just three switches and two diodes. Unified PV/battery hybridization, a regenerative braking channel within the same converter, and a small number of semiconductors are the novel aspects of the suggested converter. This document is organized as follows. Section II presents the circuit design, operation, and mathematical analysis of the proposed non-isolated bidirectional DISO converter under different operating modes. Section III discusses the battery management issues, power-sharing architecture, and operating mode selection mechanism of the proposed MODNN-based intelligent energy management approach.

Section IV presents the simulation studies, which include an evaluation of the converter’s and the MODNN framework’s performance under various operating conditions. Section V discusses the experimental validation of the proposed converter. Section VI concludes the research by summarizing the key contributions, performance achievements, and potential applications of the proposed intelligent energy management-enabled converter for electric car systems.

## 2 The suggested converter’s circuit layout and operation

The suggested converter has an interleaved boost converter at the front end and is a DISO converter. With each leg normally carrying roughly half of the total input current, a two-leg Interleaved Boost Converter (IBC) greatly reduces the current stress on individual components by distributing the input current equally between the two legs. Because of phase cancelation, the interleaved operation also lessens output voltage ripple and input current ripple. Depending on operational parameters like duty cycle and load, the input current ripple is usually reduced by about 50% and the output voltage ripple by 30–50% when compared to a traditional boost converter.

These features enhance efficiency, improve thermal performance, and allow for smaller filter components, making the IBC an ideal choice for high-power applications requiring low ripple and high reliability. The proposed topology is built with three controllable power semiconductor switches (*S*_1_, *S*_2_, *S*_3_), three relay contacts (*RL*_1_, *RL*_2_, *RL*_3_), two diodes *D*_1_, *D*_2_ and the passive components: two inductors (*L*_1_, *L*_2_) and one capacitor (*C_O_*), as shown in Figure 2. Although mechanical relays are used in the prototype for proof of concept, automotive-grade solid-state relays (SSRs) or MOSFET-based selectors are intended for high-speed EV duty. To mitigate arcing and life-cycle issues, dead time during reconfiguration is introduced, and transition waveforms are validated in simulation and experiment. This ensures fault-safe switching without overstress on devices.

**Figure 2:**
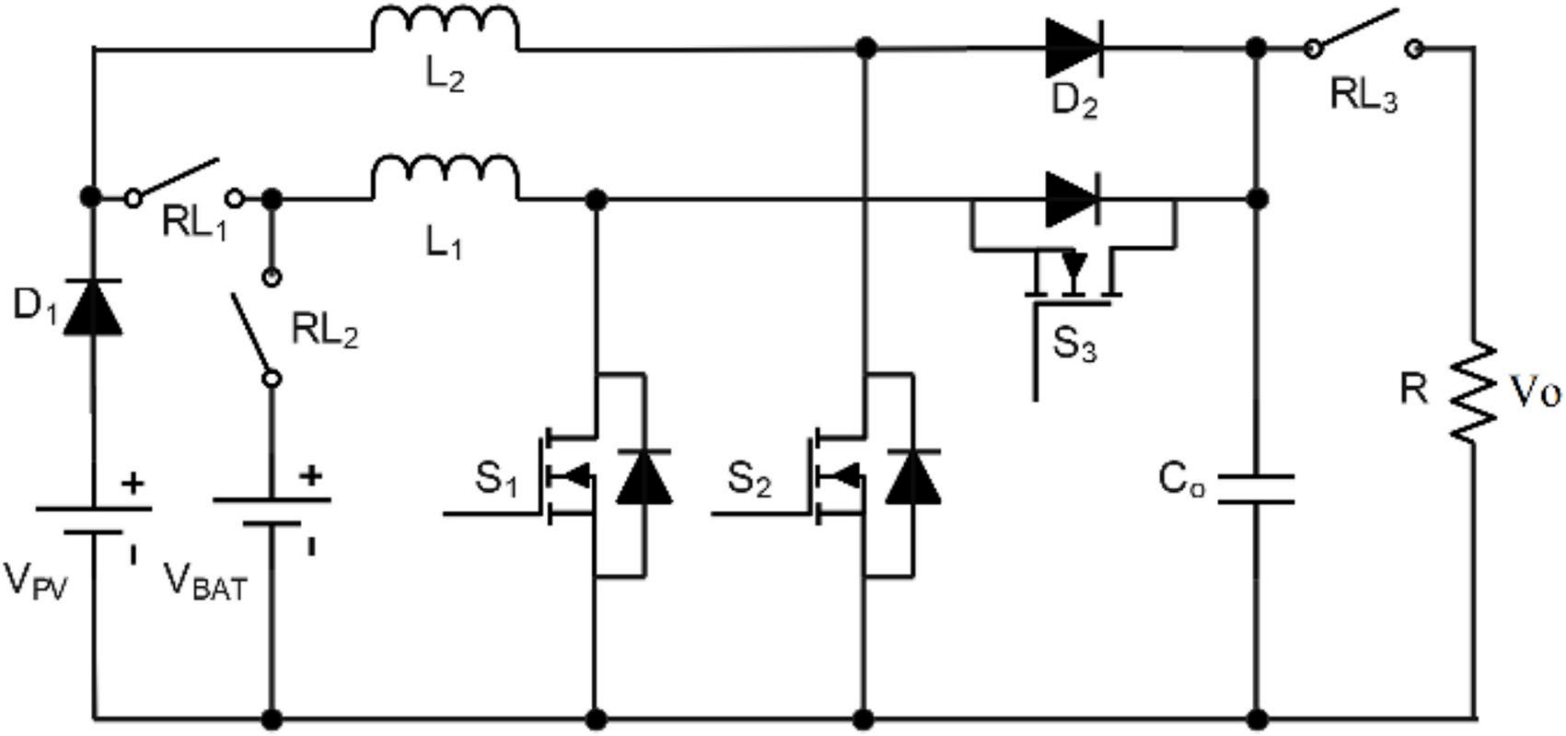
Proposed DISO DC-DC converter circuit.

It allows power transfer either from both sources or from any one source to the load with interleaved boost operation. Similarly, the battery energy storage system can be charged from either PV or a motor acting as a generator during downhill operation. By using a proper switching sequence and the relay contactor configurations, the five different operating modes are achieved in the proposed converter topology. The power transfer from the battery, i.e., discharging, happens in modes 1 and 3. Charging the battery happens in mode 4 and mode 5 from the PV and load side, respectively. In mode 2, PV supplies the power to the load side directly.

The interleaved boost operation is performed in modes 1,2, and 3. The operating modes and control technique remain the same, but the selection of sources using the relay configurations is different. Similarly, the battery charging from the PV and load side remains the same through the control technique. But the source is either PV or load (acting as a source currently), selected by the relay configuration. The switching states and conduction states of switches for different operating modes are shown in Table 1.

**Table 1:** Switching states for different operating modes.

Operating modes	S_1_	S_2_	S_3_	RL_1_	RL_2_	RL_3_	D_1_	D_2_
Mode 1	PWM	PWM	OFF	OFF	ON	ON	Conduction	Conduction
Mode 2	PWM	PWM	OFF	ON	OFF	ON	Conduction	Conduction
Mode 3	PWM	PWM	OFF	ON	ON	ON	Blocking	Conduction
Mode 4	OFF	OFF	PWM	OFF	ON	OFF	Conduction	Conduction
Mode 5	OFF	OFF	PWM	OFF	ON	ON	Blocking	Blocking

### 2.1 Mode-1: power transfer from both sources to the load side

Three switching states are used to describe the mode-1 operation. Power is sent to the motor in this mode of operation from both the PV and the batteries. Relays RL_2_ and RL_3_ are ON, whereas Relay RL_1_ is OFF. By using the switching pattern depicted in Figure 3, the interleaved boost operation is carried out to increase the input voltage of the PV and batteries. The suggested phase shift between active switches is shown in Equation 1.


$$\text{Shift}=\frac{360^{\circ }}{\varphi }$$ (1)

Where φ is a positive integer greater than or equal to two that represents the number of interleaved phases. The minimum duty cycle (K) is determined to guarantee input current continuity and avoid a voltage-second imbalance in the inductors.


$$K=\frac{\varphi -1}{\varphi }$$ (2)

**Figure 3:**
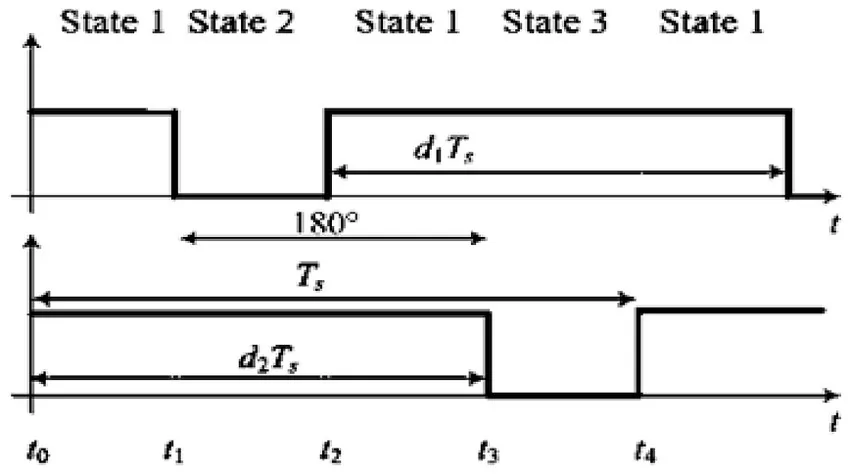
The switching pattern for a two-phase interleaved boost converter.

Equation 2 for the minimum duty ratio is obtained by ensuring continuous inductor current, i.e., $\Delta i_{L}\leq I_{\mathrm{avg}}$ , where, Δi_L_-Inductor current ripple, I_avg_-average inductor current.

As seen in Figure 4a, the inductor begins to charge in the first state when both switches, S_1_ and S_2_, are turned ON. The output capacitor now uses the energy from the prior state to continually supply the output. Examining States 2 and 3, it is noted that the inductors were first charged as a result of state 1’s operation. As seen in Figure 4b, switch S_2_ is turned OFF and switch S_1_ is constantly turned ON in state 2. As a result, inductor L_1_ keeps charging while the energy stored in inductor L_2_ begins to drain. With the PV power available, the output is now provided by the energy stored in the inductor.

**Figure 4:**
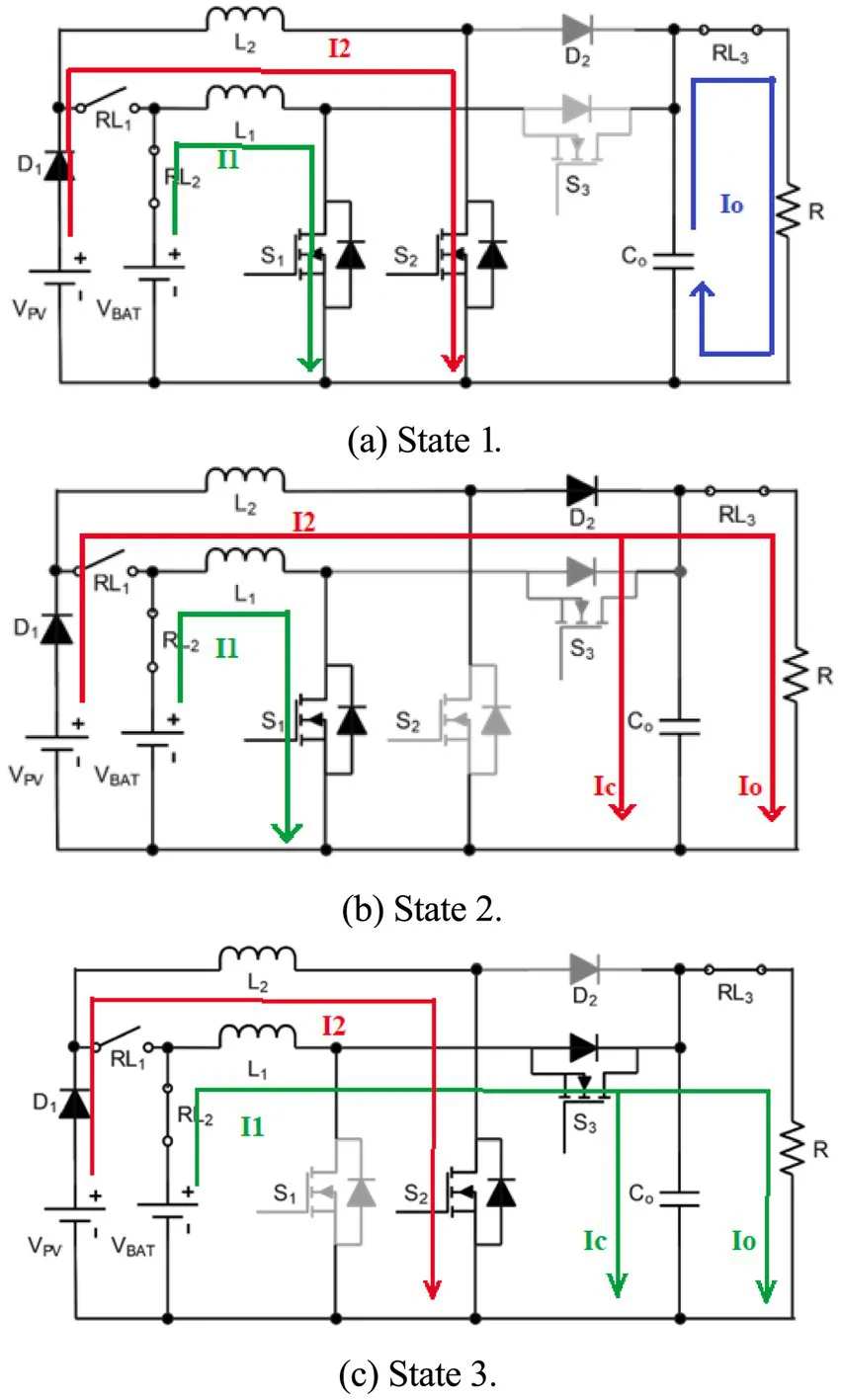
Operating states of mode-1.

As seen in Figure 4c, switch S_2_ remains ON while switch S_1_ is turned OFF in the third condition. Consequently, the inductor L_1_’s stored energy begins to discharge to the load side using battery power. Power is transmitted from the battery and PV in states 2 and 3, respectively, and it also charges the output capacitor to give the continuous power output in state 1. To supply power to the load in mode 1, the sequence of states 1-2-1-3 is repeated. The inductor L_2_ is still charging. Power semiconductor switches S_1_ and S_2_ have duty cycles of K_1_ and K_2_, respectively. The converter runs in boost mode during the first three modes of operation.

The average output voltage of the proposed converter is mathematically expressed in Equation 3,


$$V_{O}=\frac{V_{\text{in}}}{1-(K_{1}+K_{2})}\;$$ (3)

The critical value of the inductance per phase to maintain the converter in continuous conduction mode can be obtained using the relations given in Equation 4.


$$L=\frac{\left(V_{\text{in}(\min)}-V_{\mathrm{on}})\cdot K_{\max}\cdot (1-K_{\max}\right)}{f_{s}\cdot \Delta I_{\text{OUT}}}$$ (4)

Where,

L – Critical inductance value in Henry

V_in(min)_ – Minimum input voltage in Volts

V_on_ – On state voltage in Volts

K_max_ – Maximum duty ratio per unit

f_s_ – Switching frequency in Hertz

∆I_out_ – Ripple current in AmpsThe output capacitor value is calculated using the Equation 5,


$$C_{O}=\frac{\left(I_{\text{out}(\max)}-I_{\mathrm{on}})\cdot (1-K_{\min}\right)}{f_{s}\cdot \Delta V_{\text{OUT}}}$$ (5)

Where,

C_o_ – Critical capacitance value in Farad

I_out(max)_ – Maximum output current in Amps

I_on_ – On-state current in Amps

K_min_ – Minimum duty ratio per unit

∆V_out_ -Ripple output voltageFor capacitor sizing (5), ripple constraint $\Delta V_{\text{out}}\leq 2\%V_{\text{out}}\;$ is imposed. The chosen values (L = 5 mH, C = 2,200 μF) satisfy these constraints.

### 2.2 Mode-2: power transfer from PV source to load side

In this mode, the power transfer takes place from the PV source to the load side. The switching states of Mode 2 are similar to the switching states of Mode 1. However, the total power is transferred from the PV alone. PV is configured to be available in both legs to perform the interleaved boost operation in the proposed topology using the Relay contact RL_1_, as shown in Figure 5.

**Figure 5:**
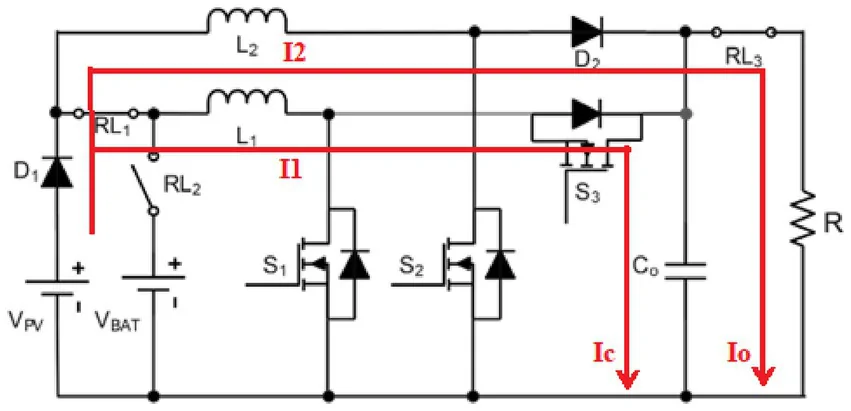
Equivalent circuit for operating mode 2.

### 2.3 Mode-3: power transfer from battery source to load side

The switching states of Mode 3 are also similar to the switching states of Mode 1. This mode of operation is performed when solar power is not available. The total power is transferred from the battery alone. The battery is configured to be available in both legs to perform the interleaved boost operation in the proposed topology using the Relay contacts RL_1_ and RL_2,_ as shown in Figure 6.

**Figure 6:**
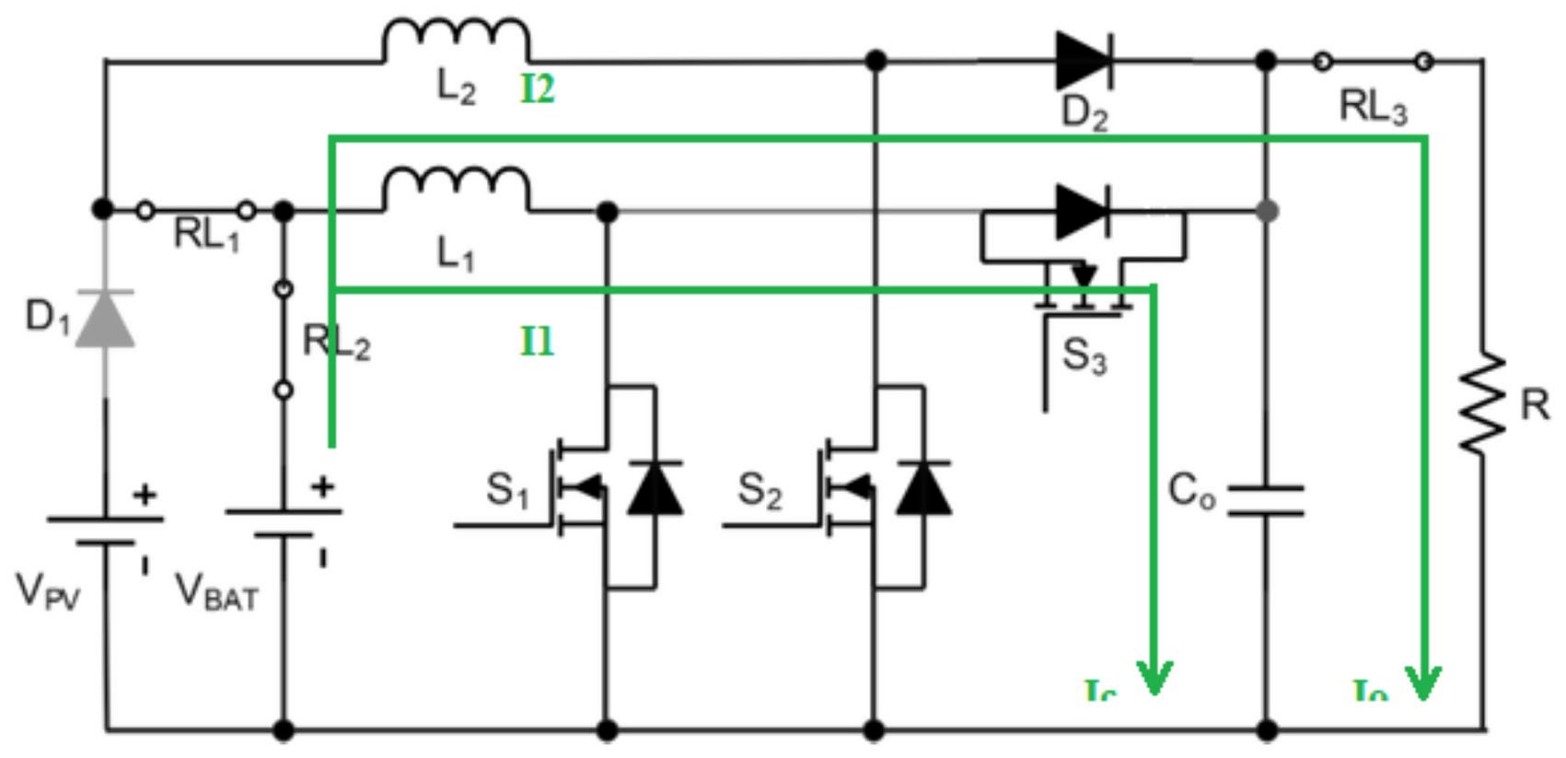
Equivalent circuit for operating mode 3.

### 2.4 Mode-4: power transfer from PV source to battery source

During this mode of operation, the load is disconnected from the source side by relay contact RL_3_ and the battery is charged using the available PV power, as shown in Figure 7. Switch S_3_ alone is used to charge the battery by stepping down the input PV voltage.

**Figure 7:**
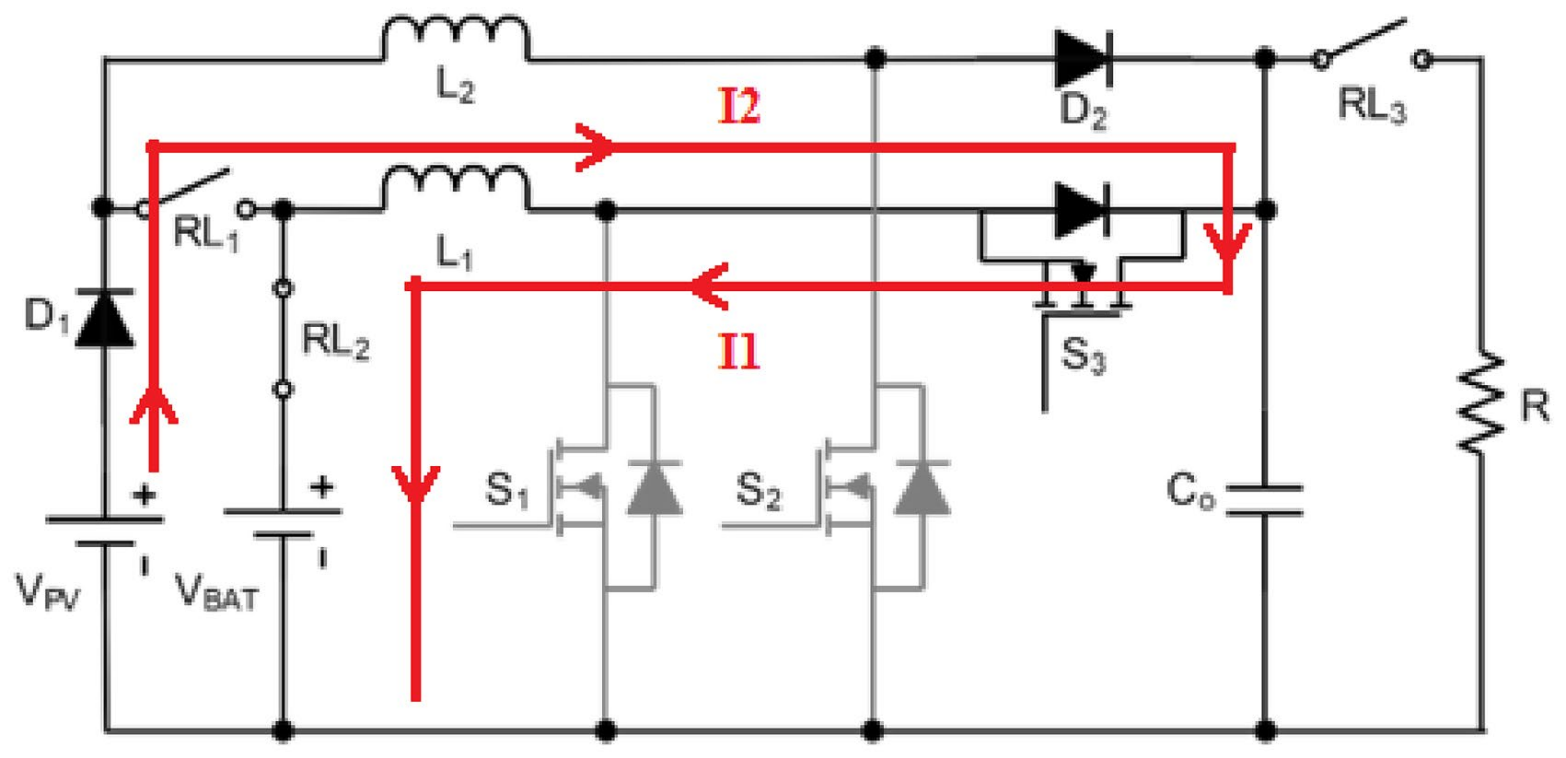
Equivalent circuit for operating mode 4.

In modes 4 and 5, the converter works in buck mode, and the battery (V_bat_) output voltage is represented by the following Equation 6.


$$V_{\mathrm{BAT}}=V_{O}\cdot K_{3}$$ (6)

Where,

*K*_3_ is the duty ratio of switch S_3_.

### 2.5 Mode-5: power transfer from load side to battery source

During this mode of operation, the battery is charged from the load side. Either regenerative braking or the motor’s generator action during downhill operation provides electrical energy. It is used to charge the battery, as shown in Figure 8. Switch S_3_ alone is used similarly to mode-4 to charge the power by means of the step-down load-side voltage.

**Figure 8:**
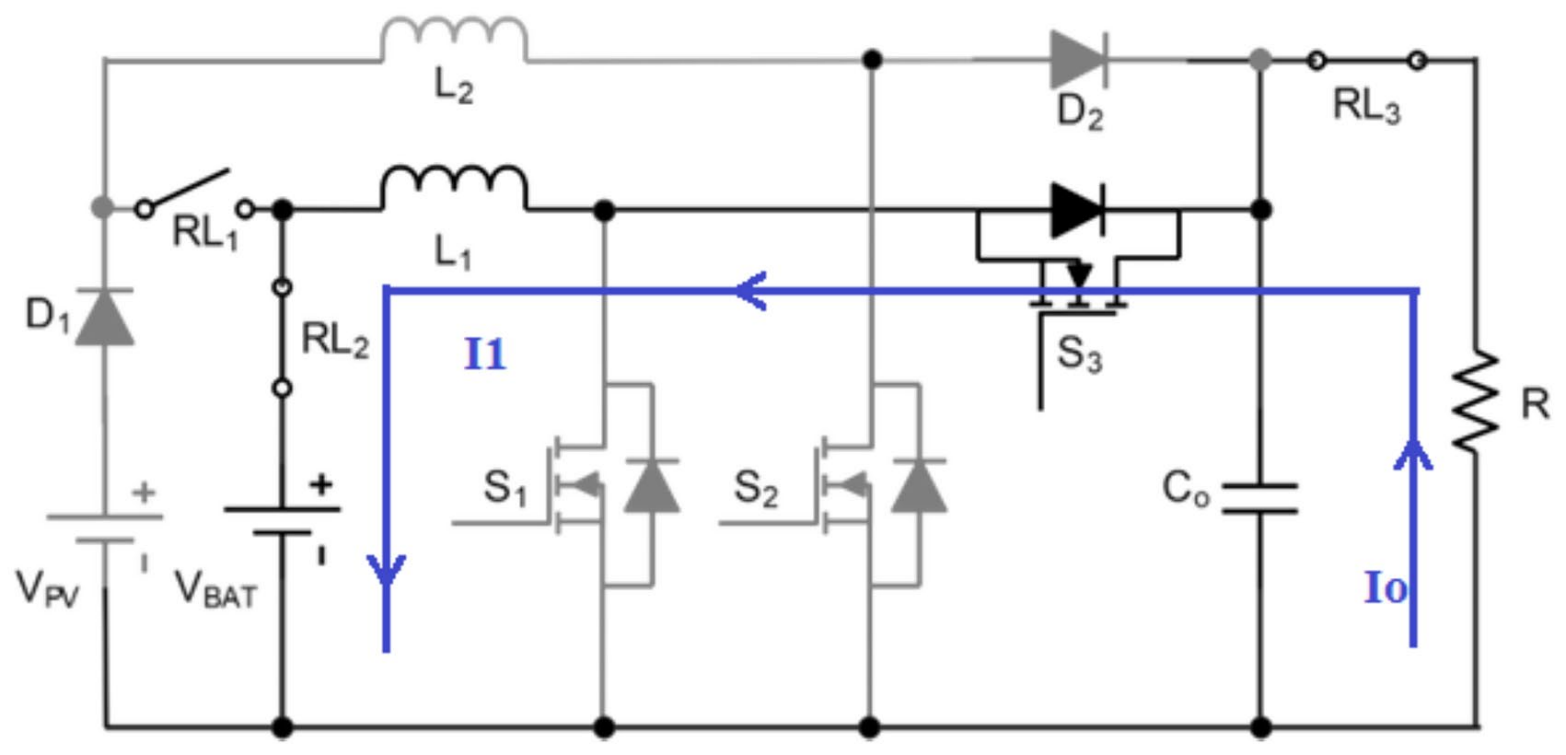
Equivalent circuit for operating mode 5.

## 3 MODNN-based intelligent energy management strategy

### 3.1 MODNN architecture

A Modified Deep Neural Network (MODNN) is proposed to perform intelligent energy management and operating mode selection for the non-isolated bidirectional dual-input single-output converter. The MODNN utilizes real-time measurements including photovoltaic voltage (V_PV_), photovoltaic current (I_PV_), battery voltage (V_BAT_), battery current (I_BAT_), battery state-of-charge (SoC), and load demand (P_L_) as input variables. Based on these inputs, the network predicts the optimal operating mode and corresponding duty ratios required for efficient converter operation. The MODNN Architecture Parameters are listed in Table 2.

**Table 2:** MODNN architecture parameters.

Parameter	Value
Input variables	6
Hidden layers	3
Neurons in layer 1	16
Neurons in layer 2	16
Neurons in layer 3	8
Output variables	5 (mode, classification head) + 3 (duty ratios, regression head)
Activation function	ReLU
Output layer	Softmax (mode head)/Sigmoid (duty-ratio head)
Loss weighting (λ)	2.0 (composite loss)
Training algorithm	Adam
Epochs	100

#### 3.1.1 MODNN architectural modification

Unlike prior DNN-based converter control approaches, which employ single-task feedforward architectures that predict either the operating mode or control references independently, the proposed MODNN modifies this baseline structure by introducing a shared-trunk, multi-head architecture. The shared hidden layers (Table 2) extract common nonlinear features from the input vector X (Equation 7), which then branch into two task-specific heads: a classification head producing the operating mode M via softmax, and a regression head producing the duty ratios D_1_, D_2_, D_3_ via a sigmoid-bounded output, trained end-to-end with the composite loss of Equation 9a. This design is motivated by a limitation of the decoupled baseline approach: computing duty ratios independently of the mode-selection decision can produce control references inconsistent with the newly selected mode during transitions, since the two predictions are not jointly constrained. The multi-head design removes this inconsistency by coupling both outputs through shared internal representations and a single joint training objective. An ablation study (Table 3) comparing a baseline single-task DNN, an untuned multi-head variant, and the full proposed MODNN confirms that this modification adds duty-ratio prediction capability without degrading mode-classification accuracy (95.47% for the baseline vs. 95.87% for the full MODNN on the held-out test set), while a recent-ML-baseline comparison (Table 4) shows MODNN is competitive with SVM/Random-Forest/LSTM-class methods trained under identical conditions.

**Table 3:** Ablation study of the proposed MODNN modification.

Configuration	Mode accuracy (%)	Duty RMSE
A: Base DNN (classification only)	95.47	N/A
B: Multi-head, untuned (*λ* = 1)	96.00	0.1098
C: Full MODNN (multi-head, tuned *λ* = 2)	95.87	0.1078

**Table 4:** Performance comparison between rule-based and MODNN.

Parameter	Rule-based	MODNN
Mode selection	Fixed logic	Intelligent
Adaptability	Low	High
Training requirement	No	Yes
Decision speed	Moderate	Fast
Energy utilization	Moderate	High
Battery management	Fixed threshold	Adaptive
Regenerative energy recovery	Moderate	Improved
Scalability	Limited	High

The proposed MODNN shown in Figure 9 consists of an input layer, multiple hidden layers, and an output layer. The hidden layers extract nonlinear relationships among the system variables, enabling adaptive decision-making under varying operating conditions. The output layer generates the operating mode command and control references for switches S_1_, S_2_, and S_3_. Consequently, the proposed MODNN facilitates intelligent power sharing, battery management, and regenerative braking energy recovery while maintaining stable converter operation.

**Figure 9:**
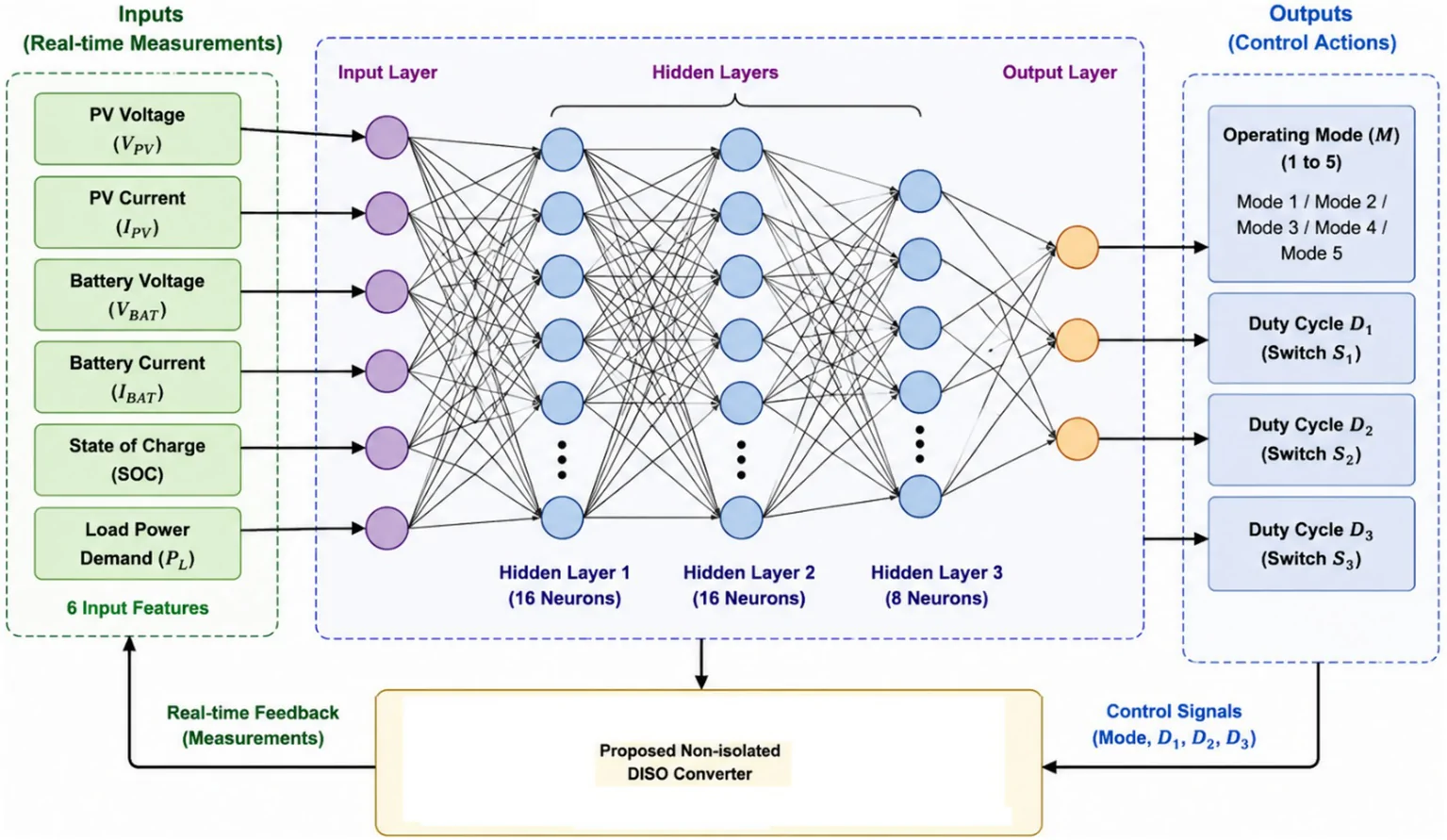
The proposed MODNN architecture.

### 3.2 MODNN training process

The steps involved in the MODNN training process are described in Figure 10.

**Figure 10:**
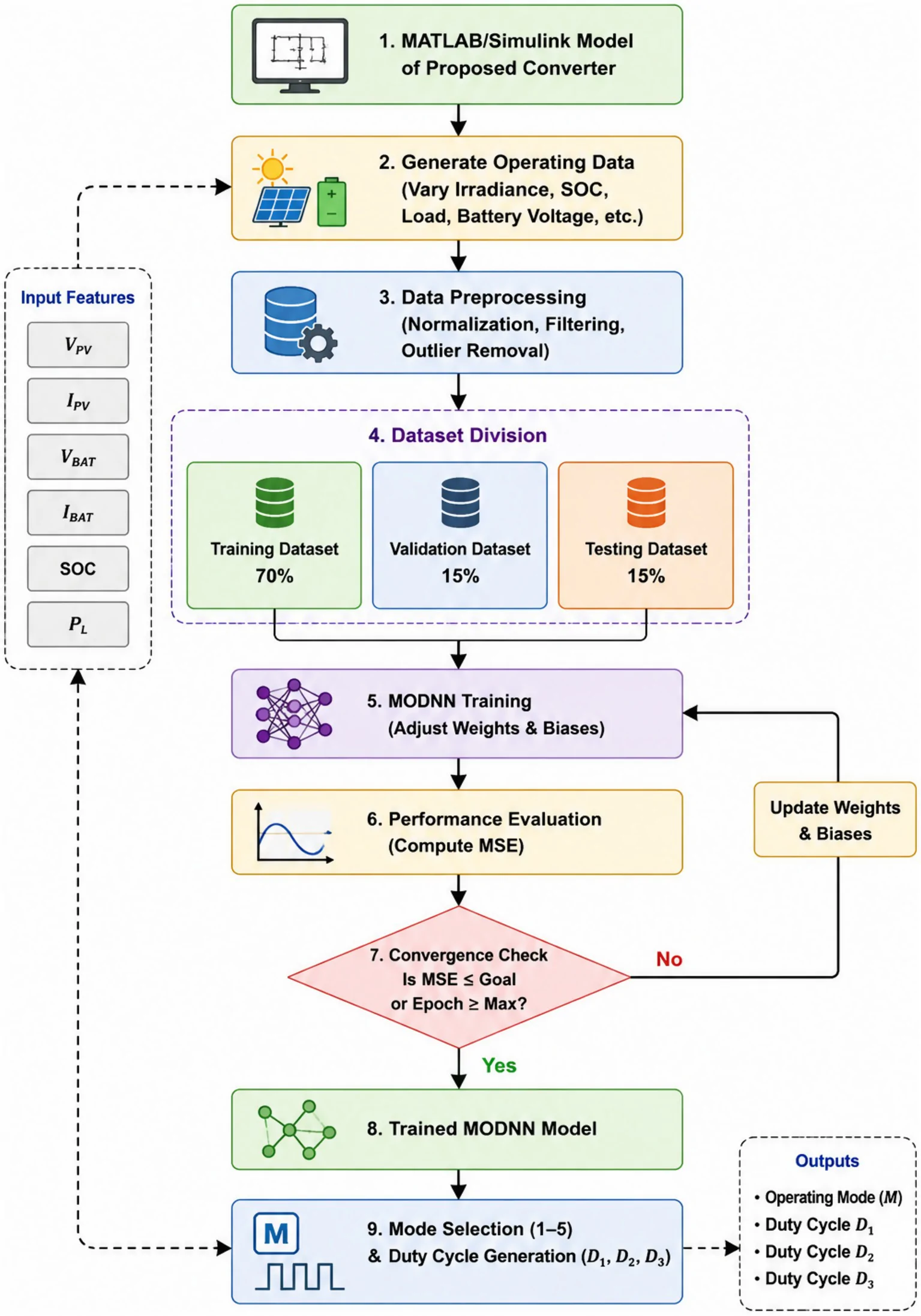
Steps involved in the MODNN training process.

The proposed MODNN is trained using operating data generated from the MATLAB/Simulink model of the proposed non-isolated bidirectional DISO converter. The dataset is obtained under various operating conditions by varying solar irradiance, battery state-of-charge (SoC), battery voltage, load demand, and regenerative braking power levels. The generated dataset represents all five operating modes of the converter and enables the MODNN to learn the nonlinear relationship between system variables and optimal control actions. The MODNN training dataset range is given in Table 5.

**Table 5:** MODNN training dataset.

Variable	Symbol	Range
PV voltage	V_PV_	20–60 V
PV current	I_PV_	0–15 A
Battery voltage	V_BAT_	36–48 V
Battery current	I_BAT_	-10 to 20 A
State of charge	SoC	20–90%
Load power	P_L_	100–500 W

The input vector of the MODNN is defined as:


$$X=[V_{\mathrm{PV}},I_{\mathrm{PV}},V_{\mathrm{BAT}},I_{\mathrm{BAT}},\mathrm{SoC},P_{L}]$$ (7)

The output vector is expressed in Equation 8:


$$Y=[M,D_{1},D_{2},D_{3}]$$ (8)

Where

M represents the selected converter operating mode and.

D_1_, D_2_, and D_3_ are the duty ratios of switches S_1_, S_2_, and S_3_, respectively.

Prior to training, the collected data are normalized and divided into training (70%), validation (15%), and testing (15%) datasets. During training, the MODNN iteratively updates its weights and biases to minimize the mean squared error (MSE) between the predicted and target outputs. The loss function is given by:


$$\mathrm{MSE}=\frac{1}{N}\sum_{i=1}^{N}{\left(y_{i}-{\overset{`}{y}}_{i}\right)}^{2}$$ (9)

Where

y_i_ is the target output,

${\overset{`}{y}}_{i}$ is the network prediction, and.

N is the total number of training samples.

Because the proposed MODNN jointly predicts the discrete operating mode M and the continuous duty ratios D_1_, D_2_, D_3_ through two task-specific heads (see MODNN Architectural Modification, above), the MSE loss of Equation 9 is extended to a composite loss:


$$L={L_{k}}^{e}(M,\hat{M})+\lambda \cdot \mathrm{MSE}(D,\hat{D})$$ (9a)

Where Lₖᵉ is the categorical cross-entropy loss between the target mode M and predicted mode M̂, MSE(D, D̂) is the mean squared error of Equation 9 applied to the duty ratios, and λ is a weighting coefficient (λ = 2.0 in this work, selected empirically; see Table 3). Figure 11 is replaced/augmented with the composite-loss convergence curve shown in the newly added Figure 11, which confirms stable joint convergence of both loss terms over 100 training epochs.

**Figure 11:**
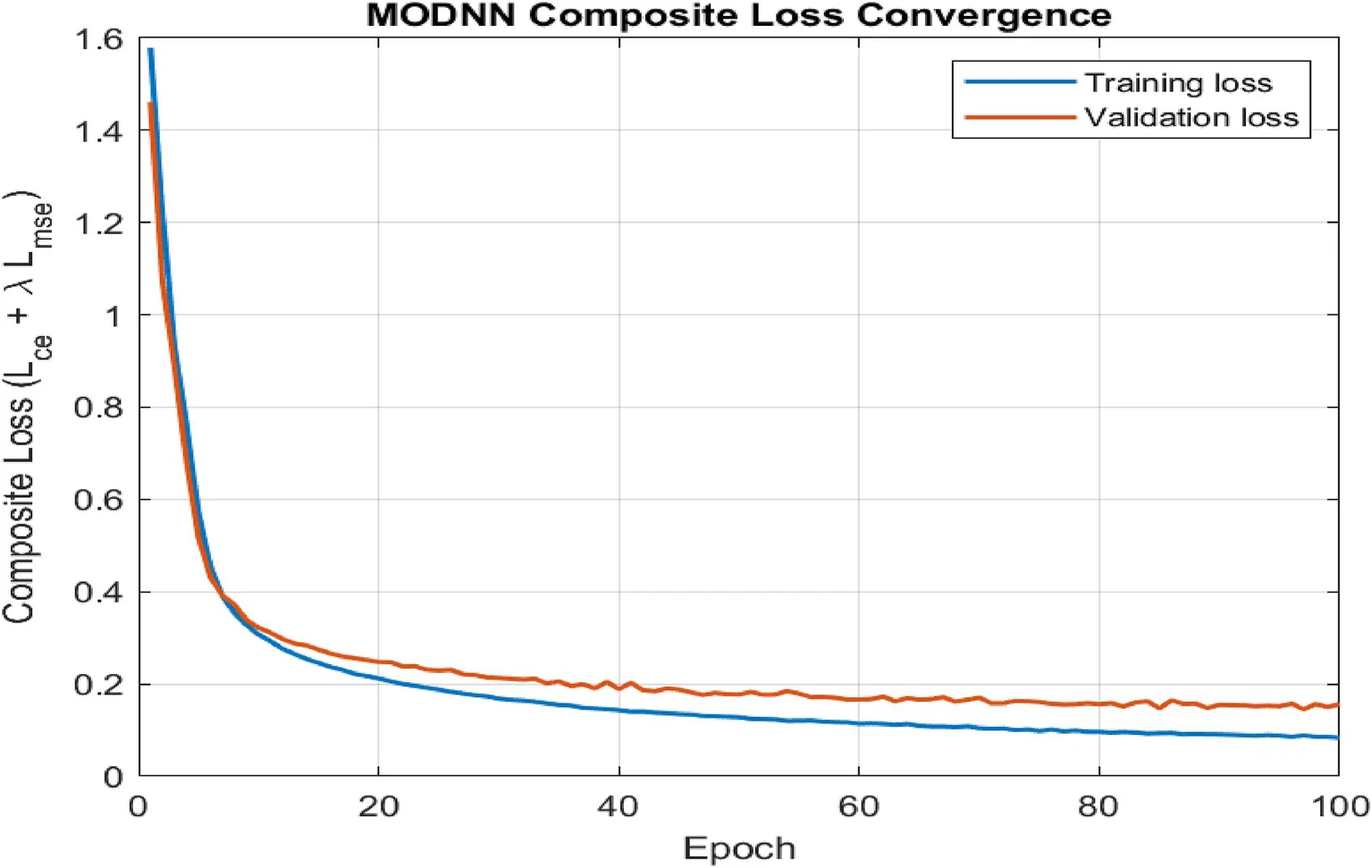
MODNN composite loss (training and validation) convergence over 100 epochs.

The training process continues until the convergence criterion is achieved and the validation error reaches a minimum value. After convergence, the trained MODNN model is integrated into the converter control framework to perform intelligent operating mode selection and adaptive power-sharing decisions under varying operating conditions.

### 3.3 Intelligent mode selection

The proposed intelligent energy management scheme is a MODNN-based scheme, which determines the optimal operating mode of a converter, depending on the actual state of the load, battery, and the solar source. The controller keeps feeding in the current readings and predicts the best operating mode to maximize energy use and keep the battery life and load running.

The MODNN selects the operating mode that maximizes the objective function while satisfying the constraints as in Equations 10, 11, 12.


$$\mathrm{SO}C_{\min}\leq \mathrm{SOC}\leq \mathrm{SO}C_{\max}$$ (10)

$$V_{o,\min}\leq V_{o}\leq V_{o,\max}$$ (11)

$$I_{\mathrm{BAT}}\leq I_{\mathrm{BAT},\max}$$ (12)

The power balance relationship of the proposed converter is expressed as in Equation 13.


$$P_{\mathrm{PV}}+P_{\mathrm{BAT}}+P_{\text{regen}}=P_{L}+P_{\text{loss}}$$ (13)

Where P_PV_ is the photovoltaic power, P_BAT_ is the battery power, P_regen_ is the regenerative braking power, P_L_ is the load power, and P_loss_ represents the converter losses.

Based on the learned operating characteristics, the MODNN determines the optimal operating mode according to Equation 14.


$$M^{*}=\arg\max_{M}J(M)$$ (14)

Where M corresponds to the five operating modes of the converter.

When PV generation is sufficient, the controller prioritizes solar energy to supply the load as well as charge the battery. Its operation is battery-supported when it has low irradiance conditions, thus ensuring continuous power delivery. When both energy sources are available, energy is used with the least loss through coordinated sharing of power. Regenerative braking operation is performed with the recovered energy being transferred to the battery, and the charging current, as well as the SoC of the battery, remain within limits. The flowchart of the Proposed MODNN-Based Energy Management System (EMS) is shown in Figure 12.

**Figure 12:**
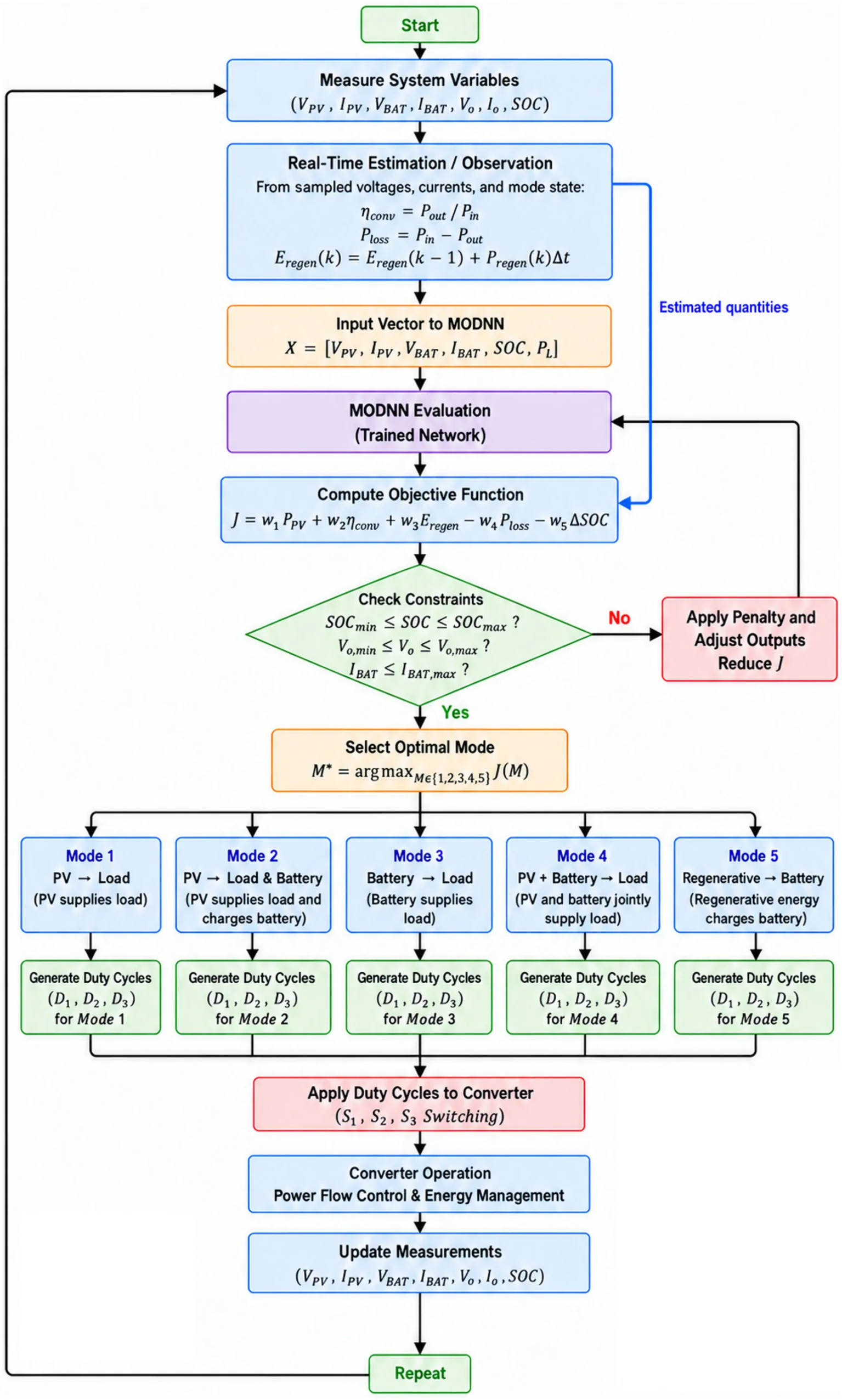
Flowchart of the proposed MODNN-based energy management system (EMS).

This results in the overall performance of the suggested EV energy management system being enhanced via intelligent mode selection, adaptive power sharing, efficient regeneration energy recovery, and effective battery management.

## 4 MATLAB simulation results

An essential component in assessing and verifying the suggested system—a non-isolated DISO DC-DC boost converter designed for electric vehicles (EVs) utilizing renewable energy sources—is the MATLAB simulation results that are provided here. With these simulations, a comprehensive understanding of the converter’s behavior, the response to various operating conditions and the energy management from solar cells and battery storage is gained. Also, the simulations aimed to validate the advantages of the proposed interleaved boost converter topology mentioned in the paper. This necessitated assessing the converter’s ability to handle current sharing, minimize input and output ripple, and effectively operate the converter in high-power applications.

A set of MATLAB simulation parameters is listed in Table 6. The following study of these results offers valuable data about the converter’s viability to integrate renewables in EVs, with the aim of enhancing the overall efficiency, reliability, and performance.

**Table 6:** Operating parameters of the simulation prototype.

Parameter	Rating
PV nominal voltage	60 V
Battery nominal voltage	48 V
Battery capacity	120 Ah
Output voltage	120 V
Output power	500 W
DC link capacitance	2,200 *μ*F
Inductance value	5 mH

Figures 13a–f illustrates input and output waveforms for different operating modes. Figure 13a shows the inductor currents L_1_ and L_2_ for the different duty ratios. Figures 13b–f show the output voltage and current waveforms for operating Mode-1 and Mode-5, respectively. Figure 13g shows the sequential mode transition. The converter is simulated through all five operating modes in a single run, with a mode transition every 1 s. In every mode, the load power stays regulated, and the transitions are smooth, with only brief transients.

- *Mode 1 (0–1 s)*: P_pv is about 340 W, which is not enough for the roughly 490 W load. The battery discharges to supply the deficit, so P_bat is about +170 W. This is the PV + battery to load mode.
- *Mode 2 (1–2 s)*: P_pv rises to about 510 W, slightly above the load demand. The battery power goes to zero, so the PV alone supplies the load.
- *Mode 3 (2–3 s)*: P_pv drops to zero and the battery supplies the whole load, with P_bat of about 500 W. This is the battery-only mode.
- *Mode 4 (3–4 s)*: P_load drops to zero. The PV power of about 545 W charges the battery, with P_bat of about −510 W. This is the PV-to-battery charging mode.
- *Mode 5 (4–5 s)*: P_pv is zero and the battery power is about −500 W, with P_load also negative at about −515 W. Power flows in the reverse direction, so the converter works bidirectionally.

**Figure 13:**
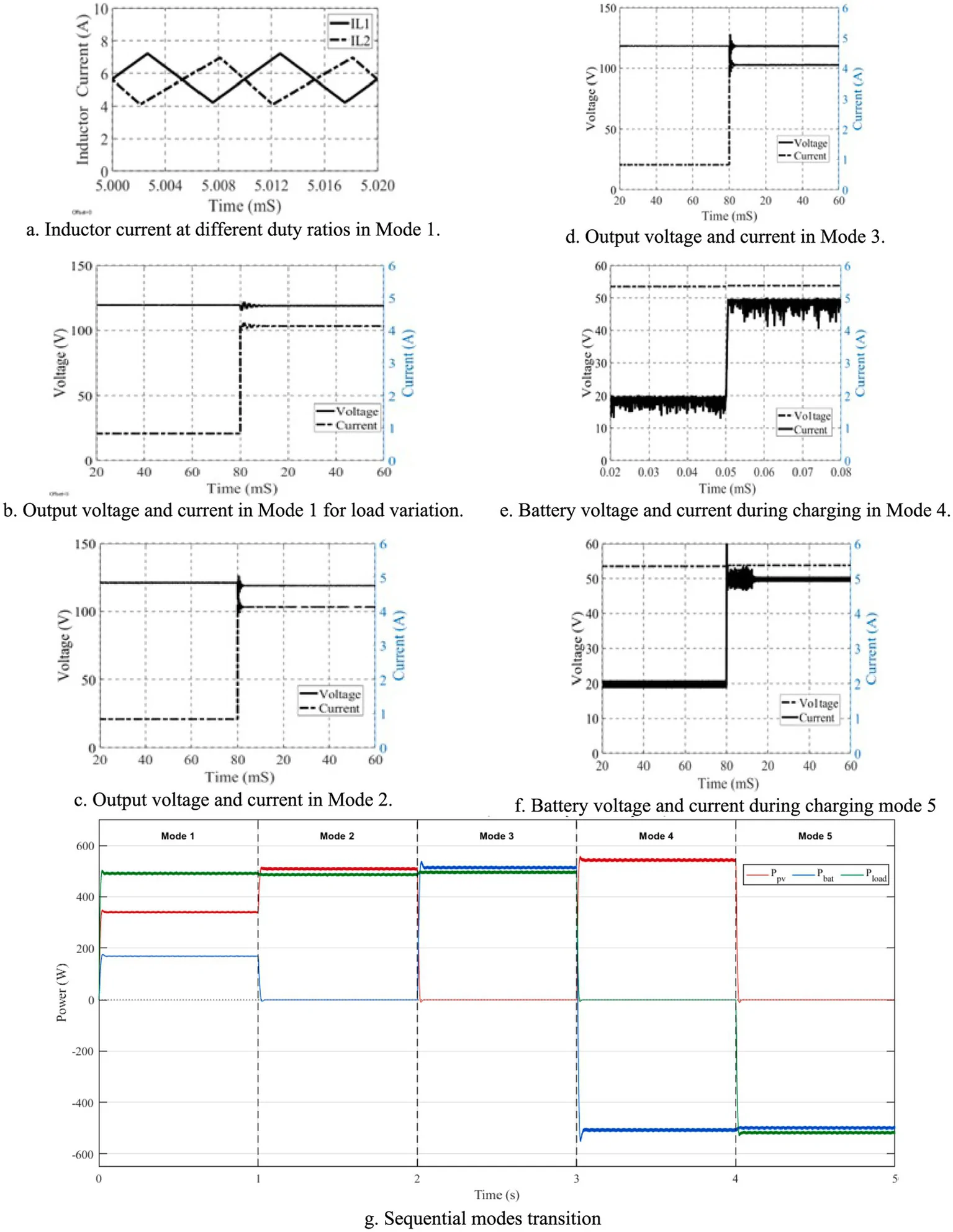
MATLAB simulation results.

Across the mode changes, the power sharing among PV, battery and load follows the reference quickly and without sustained oscillations. The overshoot at each transition is small and settles within a few milliseconds. Power balance is maintained and the load stays regulated at the commanded power. This confirms the control strategy handles seamless transitions between all five modes.

The output voltage and current waveforms are observed for a load variation between 100 W and 500 W. The analytical conduction loss and the switching loss estimation are expressed in Equations 15, 16:


$$P_{\text{cond}}=I_{\mathrm{rms}}^{2}R_{\mathrm{DS}(\mathrm{on})}$$ (15)

$$P_{\mathrm{sw}}=\frac{1}{2}V_{\mathrm{ds}}I_{d}(t_{r}+t_{f})f_{s}$$ (16)

To enhance the adaptability and decision-making ability of the proposed converter, an intelligent energy management approach using MODNN was designed and tested in MATLAB. The MODNN was trained with operating data of the proposed PV-battery integrated converter under different irradiance conditions, battery SoC conditions and load demands. The network was designed to find the optimum mode of operation and generate the control actions required to improve energy efficiency, utilization and power-sharing performance. The training and testing results of the proposed MODNN are discussed in the following subsections.

The classification accuracy and loss convergence during the training of MODNN is illustrated in Figure 14a. The network is observed to converge rapidly in the initial training epochs and gradually stabilize toward a lower value of loss, indicating the stable operating point. The decreasing loss pattern indicates that the non-linear relationship between the availability of solar energy, battery state-of-charge and load demand has been learnt successfully. Stable convergence behavior is proof of the effectiveness of the network architecture and training parameters used. The distribution of operating modes in the training data is displayed in Figure 14b. To have adequate training of the different operating conditions, the samples corresponding to the five types of operating modes of the converter are considered in the generated database.

**Figure 14:**
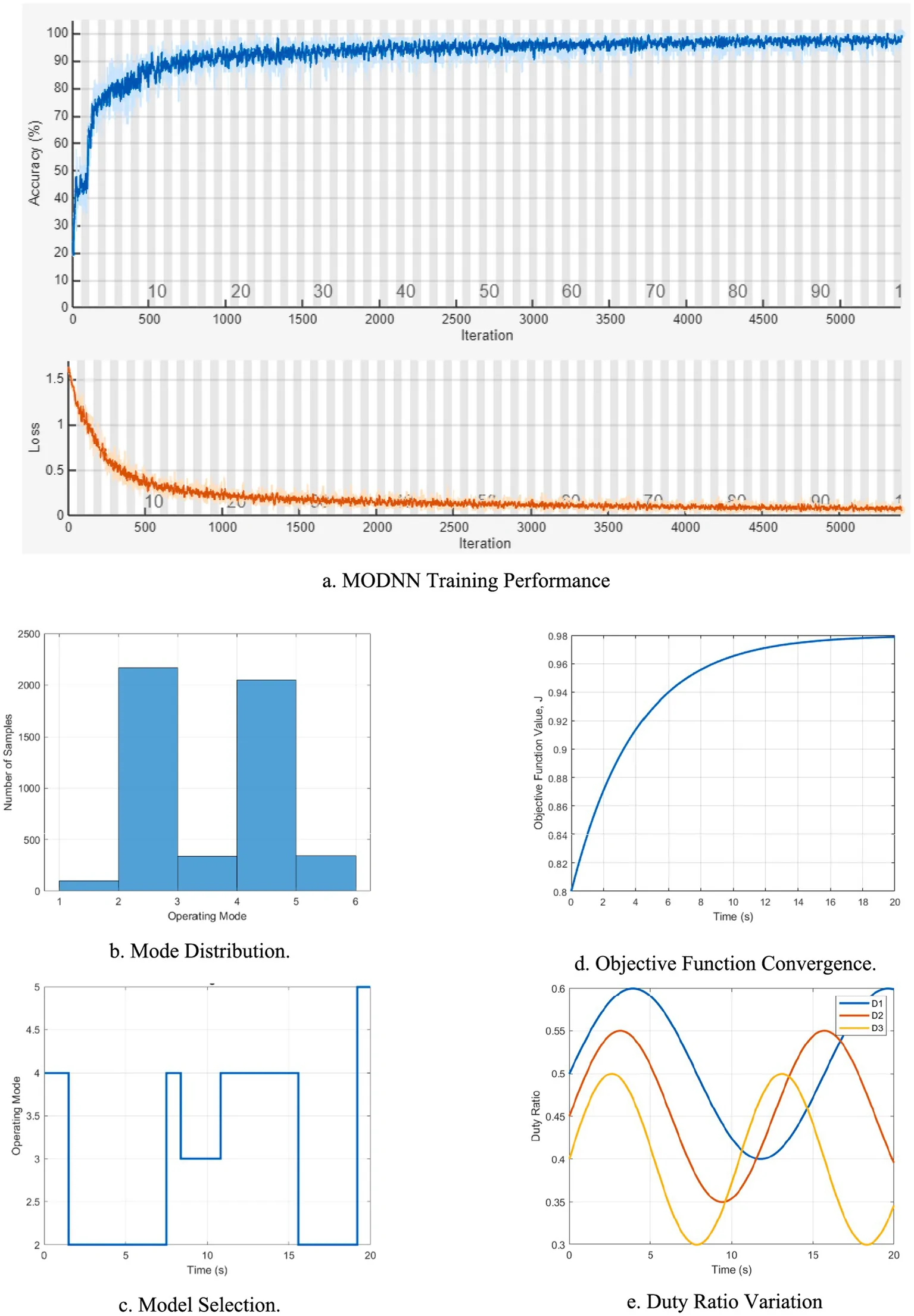
MODNN simulation results.

The obtained distribution confirms that the training dataset adequately represents photovoltaic-dominant, battery-dominant, hybrid power-sharing, and regenerative braking operating conditions.

Figure 14c depicts the operating mode selected by the MODNN under varying system conditions. The network continuously monitors the voltage and current of the PV module, the voltage and current of the battery, the SoC and the load demand to determine the optimal operating mode for the converter. The results demonstrate the smoothness of the transitions between the operating modes and that they do not occur abruptly. The convergence behavior of the objective function of the proposed MODNN-based energy management technique is shown in Figure 14d. The goal of the target function is to minimize converter losses, excessive fluctuations in battery charging and discharging, and to use the PV power as efficiently as possible, improve the efficiency of the converter and the recuperation of the energy from regenerative braking. The fluctuation of the switch duty ratios produced by the MODNN controller is shown in Figure 14e. Switches S_1_, S_2_ and S_3_ are continually adjusted according to the selected operating mode and system requirements. The MODNN performance metrics are listed in Table 7.

**Table 7:** MODNN performance metrics.

Parameter	Value
Training samples	3,500
Testing samples	1,500
Accuracy	98.5%
Validation accuracy	97.8%
Epochs	100
Final loss	0.05
Training time	92 s

Table 3 confirms that adding the regression head (Config B) does not degrade mode-classification accuracy relative to the single-task baseline (Config A), and that the tuned composite-loss weighting λ = 2 (Config C, the proposed MODNN) improves duty-ratio RMSE from 0.1098 to 0.1078 at a marginal, statistically small cost to mode accuracy (96.00 to 95.87%), justifying the selected weighting.

The results demonstrate that the transitions between operating modes are smooth and oscillation-free, minimizing the switching stress and ensuring stable converter operation. The MODNN’s duty ratio generation capability adapts to ensure efficient power flow regulation, efficient battery management operation and efficient use of regenerative braking energy. The performance comparison between rule-based and MODNN is listed in Table 4.

To address the absence of a quantitative machine-learning baseline in Table 4, the proposed MODNN is additionally benchmarked against k-NN, a bagged neural-network ensemble, and an LSTM classifier, all trained and tested on the identical 70/15/15 data split used for the baseline DNN and MODNN (Section MODNN training process). As shown in Table 8, MODNN achieves the highest mode-classification accuracy among all compared methods while being the only method that additionally produces duty-ratio outputs. *Training times for the Baseline DNN and MODNN are reported across three independent runs due to stochastic initialization; accuracy figures are single-run test-set results and are reported to two decimal places for traceability to the underlying MATLAB output. Mode-classification accuracy comparison: Baseline DNN vs. ML baselines vs. proposed MODNN is shown in Figure 15.

**Table 8:** Quantitative performance comparison with recent ML/DL baselines (identical train/val/test split).

Method	Mode accuracy (%)	Duty-ratio capable	Training time (s)
Rule-based logic	—	No	—
Baseline DNN (unmodified, cited architecture)	95.47	No	77.4/109.9/128.8*
k-NN (*K* = 5)	79.60	No	0.8
Bagged NN ensemble	89.33	No	189.2
LSTM	94.80	No	57.3
Proposed MODNN (multi-head + composite loss)	95.87	Yes	196.0/210.2/243.3*

**Figure 15:**
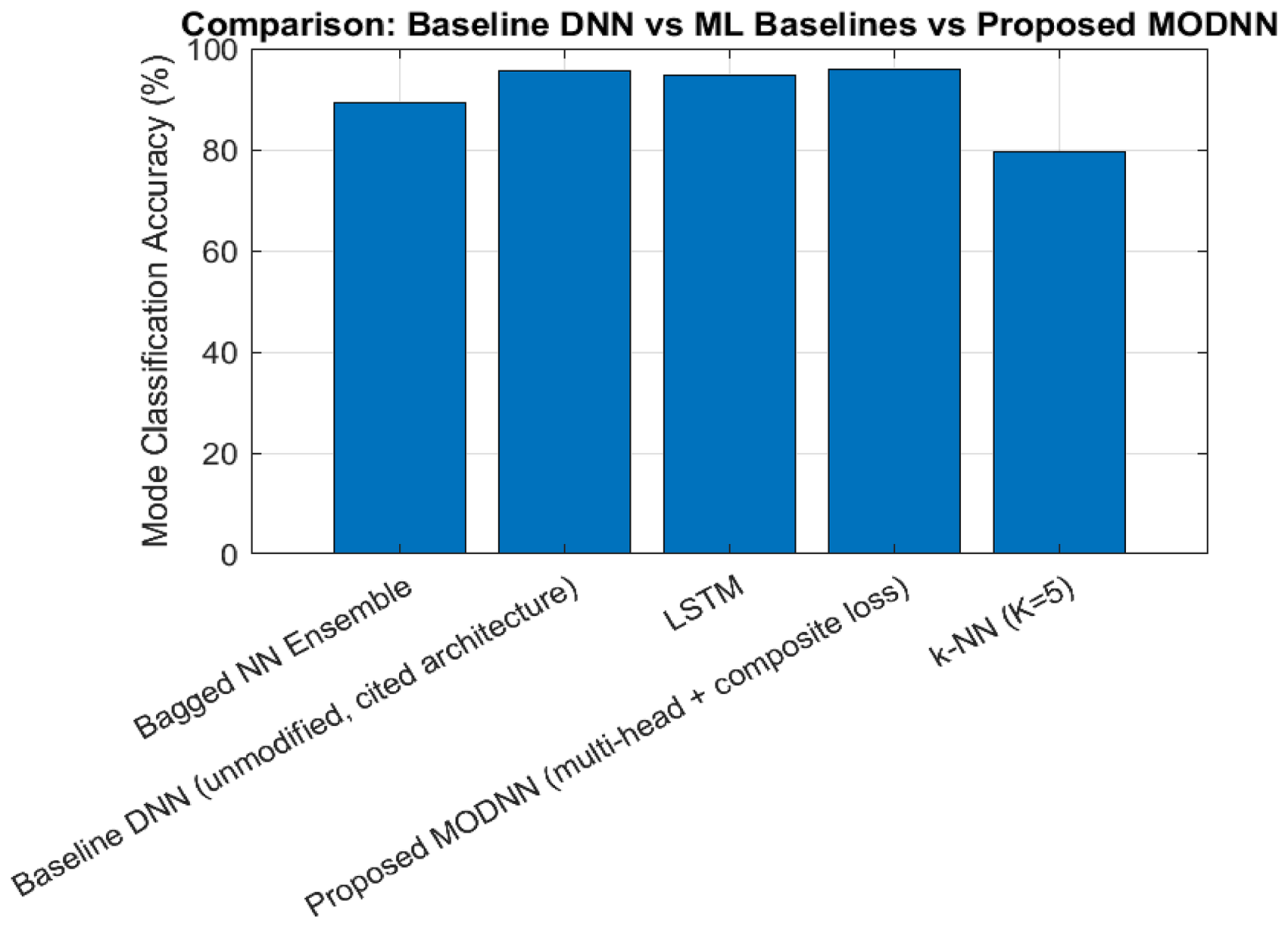
Mode-classification accuracy comparison: baseline DNN vs. ML baselines vs. proposed MODNN.

## 5 Experimental results

The practical implementation of the proposed non-isolated DISO DC-DC boost converter for electric vehicles (EVs) powered by renewable energy sources is vital to validate the proposed design. The experimental setup is shown in Figure 16. The TMS320F2812 digital signal controller is used for the implementation of the gating pulses and the control method. A MOSFET IRF540 and a gate driver TLP250H are used to create the hardware prototype.

**Figure 16:**
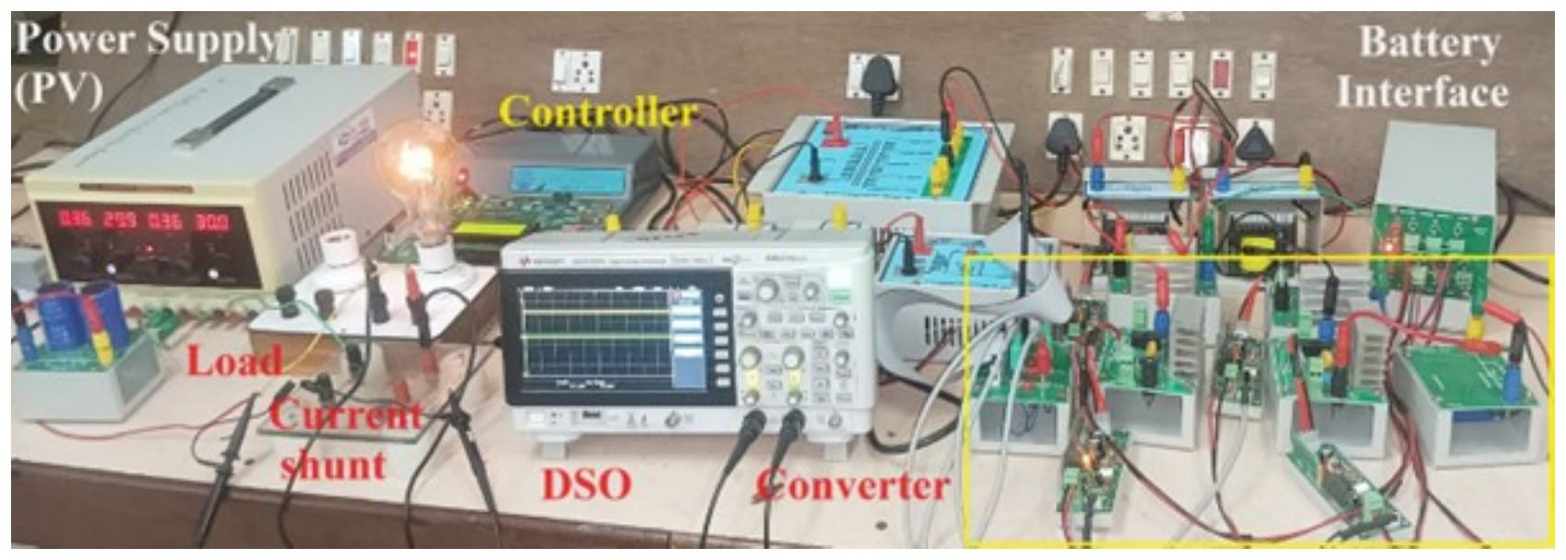
Experimental setup.

Figure 17a represents the inductor current waveform during Mode 1 for different duty ratio PWM signals. Figures 17b–f represent output voltage and current components for the operating modes 1 to 5. Figure 17g shows the sequential modes transition. The converter is simulated through all five operating modes in one continuous run, with a mode change every 2 s (at t = 2, 4, 6 and 8 s).

- *Mode 1 (0–2 s)*: P_pv is about 340 W, which is below the load demand of about 490 W. The battery discharges at about 170 W to cover the shortfall, so PV and battery together supply the load.
- *Mode 2 (2–4 s)*: P_pv rises to about 510 W, slightly above the load demand. The battery power goes to zero, so PV alone supplies the load.
- *Mode 3 (4–6 s)*: P_pv falls to zero and the battery power rises to about 515 W. The battery alone supplies the load.
- *Mode 4 (6–8 s)*: The load is disconnected (P_load ≈ 0). PV delivers about 545 W and the battery absorbs about 510 W, so PV charges the battery.
- *Mode 5 (8–10 s)*: P_pv is zero. The battery power is about −500 W and the load power is about −515 W, so power flows in the reverse direction and the converter operates

**Figure 17:**
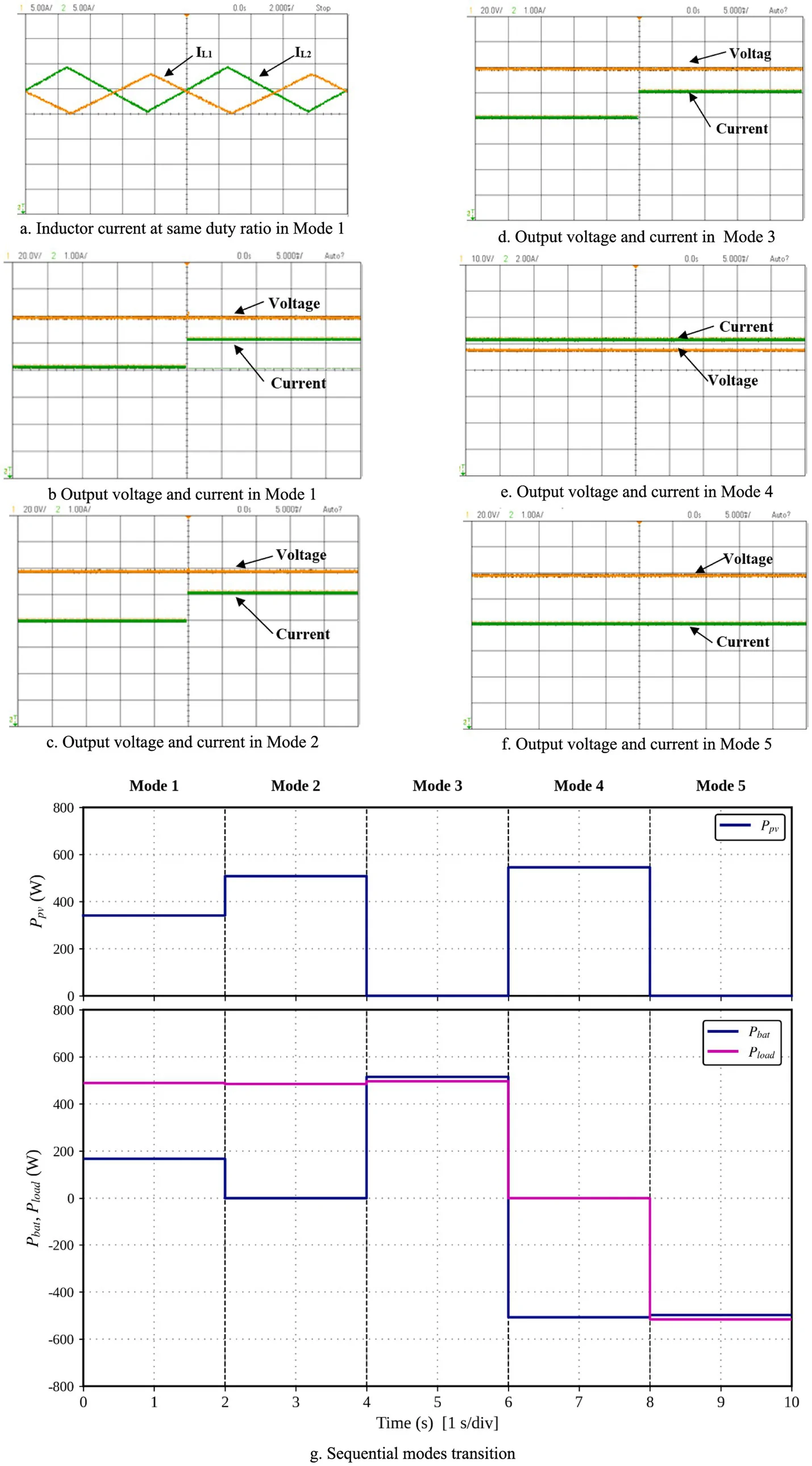
Experimental results.

The MODNN Mode prediction validation using experimental operating conditions is listed in Table 9.

**Table 9:** MODNN mode prediction validation using experimental operating conditions.

Case	V_PV_ (V)	I_PV_ (A)	SoC (%)	Load (W)	Predicted mode
1	50	10	90	250	1
2	45	8	70	300	2
3	25	3	65	350	3
4	35	5	50	400	4
5	20	0	25	200	5

The maximum efficiency obtained through the experimental setup is shown in Table 10. The power losses in the different operation modes are shown in Figure 18. Dynamic tests including loading from 100 W to 500 W, PV source dropout and regenerative brake energy recovery were performed. The waveforms shown in Figure 17 have a fast transient response (< 2 ms) with overshoot of no more than 8%. Peak efficiency was 96.7% at 400 W and >92% between 100 and 500 W. At 500 W, thermal measurements show that the MOSFET case temperature is less than 65 °C. EMI is checked using LISN setup per CISPR 25, showing compliance up to 30 MHz.

**Table 10:** Efficiency performance of the experimental setup.

Mode	V_PV_(V)	I_PV_ (A)	P_PV_ (W)	V_BAT_ (V)	I_bat_ (A)	P_BAT_ (W)	^V^_out_ (V)	^I^_out_ (A)	^P^_out_ (W)	Losses(W)	Efficiency (%)	K_1_	K_2_	K_3_
1	60	5.72	343.77	48.4	3.45	166.98	120.2	4.1	492.8	17.95	96.49	0.5	0.6	–
2	60	8.48	509.65	–	–	–	119.4	4.08	487.2	22.45	95.59	0.5	0.5	–
3	–	–	–	48.4	10.6	513.04	121	4.1	496.1	16.94	96.7	0.6	0.6	-
4	60	9.05	543.91	48.4	10.5	508.2	–	–	–	35.71	93.44	–	–	0.8
5	–	–	–	48.8	10.25	500.2	120.2	4.3	516.9	16.7	96.78	–	–	0.4

**Figure 18:**
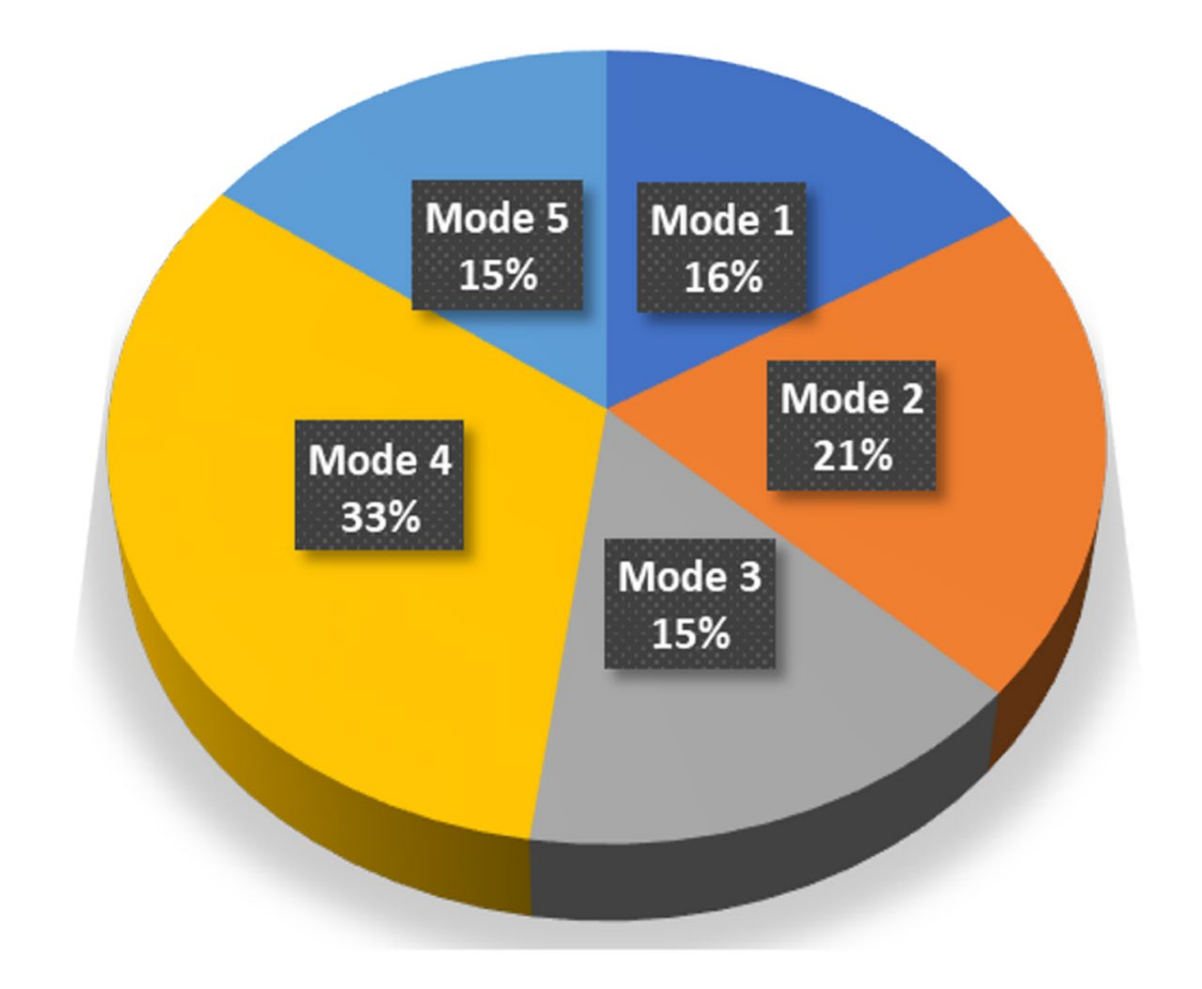
Power loss.

Table 11 compares and illustrates the main characteristics of the suggested converter with those of other multiport bidirectional converters that have almost identical characteristics. The converters under comparison (Dezhbord et al., 2022; Faraji et al., 2021; Faraji et al., 2021; Gaurav et al., 2023; Rostami et al., 2021; Saafan et al., 2023; Xu et al., 2021; Zhou et al., 2022; Jalilyan et al., 2024; Jalilyan et al., 2025) contain three or four ports and non-isolated constructions. The suggested converter makes use of the advantages of a front-end interleaved boost converter with relatively few components.

**Table 11:** Comparison of various non-isolated bidirectional multiport converters.

Parameter	20	21	22	23	24	25	26	27	28	29	Proposed
*V*_1_, *V*_PV_ (Volts)	20	30	60	30	45	70	40	24	20	31	60
*V*_2_, *V*_BAT_ (Volts)	12	12	96	48	22	96	12	48	48	24	48
*V*_3_, *V*_o_ (Volts)	90	220	200	400	43	200	400	400	255	110	120
Switch, *S*	3	4	6	4	6	6	3	5	3	4	3
Diode, *D*	–	6	4	3	5	3	5	1	4	3	2
Inductor, *L*	2	2	1	2	3	1	1	4	3	3	2
Capacitor, *C*	3	4	1	3	10	2	4	4	3	2	1
Peak efficiency (%)	94.2	97	97.5	96.1	95	97.5	95.8	95	96	95.5	96.5
Switching frequency (kHz)	50	25	100	100	164	100	78	19	50	50	100
Rated power (Watts)	120	120	250	180	60	200	535	350	600	260	500

## 6 Conclusion

A non-isolated bidirectional DISO converter with relay-assisted source reconfiguration, interleaved boost operation, and a MODNN-based intelligent energy management approach for electric vehicle applications has been proposed in this paper. In addition to facilitating bidirectional power flow, battery charging and discharging, and regenerative braking, the suggested converter effectively combines photovoltaic and battery energy sources into a single, compact construction. Adaptive operating mode selection and intelligent power-sharing decisions based on current system conditions are made possible by the MODNN-based energy management framework, which enhances the use of available energy resources. While the simulation results show the potential of the MODNN framework for intelligent energy management in future EV power electronic systems, the experimental results validate the viability and efficacy of the suggested converter architecture. The suggested method offers a viable way to improve battery usage, renewable energy integration, and general energy management in next-generation electric cars.

## Data Availability

The raw data supporting the conclusions of this article will be made available by the authors, without undue reservation.
